# Does task shifting yield cost savings and improve efficiency for health systems? A systematic review of evidence from low-income and middle-income countries

**DOI:** 10.1186/s12960-017-0200-9

**Published:** 2017-04-13

**Authors:** Gabriel Seidman, Rifat Atun

**Affiliations:** Department of Global Health and Population, Harvard T. H. Chan School of Public Health, 677 Huntington Avenue, Boston, MA 02115 United States of America

**Keywords:** Task shifting, Community health workers, Health systems, Efficiency, Cost-effectiveness, Systematic review

## Abstract

**Background:**

Task shifting has become an increasingly popular way to increase access to health services, especially in low-resource settings. Research has demonstrated that task shifting, including the use of community health workers (CHWs) to deliver care, can improve population health. This systematic review investigates whether task shifting in low-income and middle-income countries (LMICs) results in efficiency improvements by achieving cost savings.

**Methods:**

Using the PRISMA guidelines for systematic reviews, we searched PubMed, Embase, CINAHL, and the Health Economic Evaluation Database on March 22, 2016. We included any original peer-review articles that demonstrated cost impact of a task shifting program in an LMIC.

**Results:**

We identified 794 articles, of which 34 were included in our study. We found that substantial evidence exists for achieving cost savings and efficiency improvements from task shifting activities related to tuberculosis and HIV/AIDS, and additional evidence exists for the potential to achieve cost savings from activities related to malaria, NCDs, NTDs, childhood illness, and other disease areas, especially at the primary health care and community levels.

**Conclusions:**

Task shifting presents a viable option for health system cost savings in LMICs. Going forward, program planners should carefully consider whether task shifting can improve population health and health systems efficiency in their countries, and researchers should investigate whether task shifting can also achieve cost savings for activities related to emerging global health priorities and health systems strengthening activities such as supply chain management or monitoring and evaluation.

## Background

Efficient and effective health systems are critical for managing healthcare costs, addressing rising burden of disease, and providing sustainably universal health coverage. The efficiency of health spending has major implications for the health of the population. In low-income and middle-income countries (LMICs) of Africa, Asia, and the Middle East, increasing the efficiency of health spending could increase health-adjusted life expectancy by 1–2 years [1].

Human resources for health (HRH) make up a significant portion of health expenditures; in LMICs, spending on salaried health workers makes up 28.7–33.2% of total health expenditure [2]. Improving the efficiency of spending on HRH can improve the efficiency of health systems, which can free up financial and other resources and ultimately improve health coverage [3].

According to the World Health Organization (WHO), task shifting “presents a viable solution for improving health care coverage by making more efficient use of the human resources already available and by quickly increasing capacity while training and retention programs are expanded” [4]. Task shifting can produce equivalent or superior outcomes for many diseases and health interventions including non-communicable diseases [5], HIV/AIDS [6, 7], contraceptive distribution [8], and others [5, 9].

Given the high spend on HRH, the evidence for task shifting as a way to improve population health, and the prominence of task shifting on the global policy agenda, policymakers should understand the cost and efficiency implications of this approach to health systems strengthening (HSS). Therefore, our systematic review aims to answer the following question: Does task shifting result in cost savings and efficiency improvements for health systems or patients in LMICs?

To our knowledge, only one literature review has addressed a similar question so far [10]. That review found that community health workers (CHWs) are cost-effective for treating TB and select other disease areas, such as reproductive, maternal, newborn, and child health (RMNCH). Our review builds on the important initial review conducted by Vaughan et al. in three ways. First, our search strategy takes a broader scope in that it reviews other forms of task shifting besides the use of CHWs (e.g., shifting the work of physicians to nurses or the work of nurses to pharmacy technicians), which may contribute to HSS.

Second, our review looks at evidence for efficiency improvements achieved by shifting tasks from one cadre of workers to another, rather than whether an intervention using a specific type of health worker meets a cost-effectiveness threshold. Although cost-effectiveness thresholds (e.g., cost/unit of health improvement above or below a pre-defined benchmark) are an important criterion for prioritizing interventions, cost-effectiveness as measured by an actual reduction in costs without a reduction in programmatic quality is particularly salient for policymakers trying to improve the efficiency of the health system. Therefore, we review whether studies found changes in cost per input/process, output, or outcome as a result of task shifting. Whereas cost savings on inputs/processes are very likely since the wage for a lower-skilled worker will almost always be lower than that of a higher-skilled worker, cost savings on outputs and outcomes are not as guaranteed since lower-skilled workers might operate less efficiently. A reduction in cost per output or outcome can be interpreted as an improvement in efficiency and therefore a true savings to the health system (with changes in cost per outcome as the stronger indicator), but a reduction in cost per input/process can only be interpreted as an efficiency improvement if it is accompanied by the documentation of no change (or an improvement) in clinical or programmatic quality.

Third, following from the previous point, our review also captures and reports evidence of changes in programmatic or clinical quality as a result of task shifting for each included reference, which Vaughan et al. do not systematically report. Reporting programmatic quality outcomes is important for determining whether a reduction in costs actually indicates an improvement in health systems efficiency.

## Methods

This systematic review follows the criteria and methodology described in the PRISMA guidelines on systematic reviews [11].

### Search process and criteria

This search relied on an internal protocol developed by both authors, with the support of a Harvard University librarian specializing in systematic reviews. The protocol was not registered externally. We searched PubMed, Embase, CINAHL, and the Health Economic Evaluation Database. The main search that was conducted on March 22, 2016, was as follows (for PubMed), with an additional search term for LMICs, and any publication from before that data was eligible for our review:

(task shift*[tiab] OR balance of care[tiab] OR non-physician clinician*[tiab] OR nonphysician clinician*[tiab] OR task sharing[tiab] OR community care giver*[tiab] OR community healthcare provider*[tiab] OR cadres[tiab] OR “Community Health Workers”[Mesh])

AND

(“Cost Savings”[mesh] OR “Cost Benefit Analysis”[mesh] OR “Efficiency”[mesh] OR cost[tiab] OR costs[tiab] OR efficienc*[tiab] OR economies of scale[tiab] OR economies of scope[tiab] OR productivity[tiab] OR absenteeism[tiab] OR “Absenteeism”[Mesh])

We also conducted several additional searches based on a review of citation lists from relevant publications, and based on recommendations from public health researchers.

### Study selection and eligibility criteria

After conducting our search, all titles were reviewed for relevance. After excluding irrelevant titles, we read all abstracts and, when appropriate, full articles to determine the relevance of the article for our research question. In order to be included in the study, the publication had to meet the following criteria:

- Report on an effort, such as a program or policy intervention, involving task shifting of a clinical activity or health systems-related activity
- Report a comparison of program costs from the task shifted model for conducting the activity or service to a comparable activity in a model that does not involve task shifting.
- Report results from an actual intervention, rather than a computer model or simulation
- Report results from a low-income or middle-income country
- Be original research about an intervention published in a peer-reviewed format (as opposed to an editorial, literature review, opinion piece, interview, etc.)
- Have a complete article available (as opposed to just an abstract)
- Be published in English

### Data collection process

In order to extract data for this review, we piloted an Excel-based data collection tool that was used to capture results from a preliminary search, the results of which were presented at the Harvard Ministerial Leadership Program in the summer of 2016. Based on our experience with this initial process, we modified the tool accordingly and finalized a tool which collected the following information: author, year, title, publication, abstract, country, continent, description of the intervention, main indicator, result on relevant indicator, and data on programmatic quality changes resulting from the intervention. Studies were not excluded if they did not have relevant quality comparisons. Results which did not provide evidence of cost changes, such as baseline costing studies, were excluded. GS conducted a first review of all references in the search, and the list was reviewed by RA and other public health researchers in order to identify missing references or references which had been improperly included.

We also retrospectively categorized the included references based on whether the main indicator documented changes in cost per input/process, output, or outcome, using the following definitions: [12].

- Inputs/processes: resources required to conduct an activity, or a discrete activity such as a patient visit with a clinician
- Outputs: direct products of program activities, such as number of individuals treated
- Outcomes: changes in health status as a result of the program, such as number of patients cured or number of deaths averted

### Risk of bias

As with any systematic review, the references and data sources for this review contain the possibility for bias. At the level of individual references, authors are more likely to report cost data if their program resulted in cost savings, especially if costing/cost-effectiveness was not the primary purpose of the study.

Across all studies, there is also a risk of publication bias and selective reporting within studies, especially if authors more frequently chose to report positive outcomes (such as cost savings). Of course, the decision to implement task shifting in a given context would require extensive analysis of that particular intervention’s potential impact, and we caution researchers and policymakers not to interpret the findings from this review as indicative of the results that they can expect to achieve.

## Results

### Study selection

**Fig. 1:**
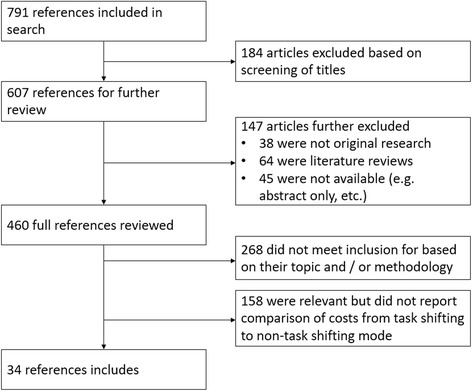
Study selection for inclusion in systematic review

We reviewed 791 articles and identified 34 references which analyzed the cost implications of task shifting in LMICs—22 in sub-Saharan Africa, eight in Asia and four in Central or South America. See Fig. 1 for the study selection for inclusion in this systematic review. Of the 32 studies included in the review by Vaughan et al., we excluded 17 and included 15, which means that our review also included an additional 19 studies not included in Vaughan et al. Of the 17 references included by Vaughan et al. that we excluded, 12 were excluded because they did not provide comparison of costs between the task shifted model and another model of care [13–24], three reported results from modeling of hypothetical programs rather than actual interventions [25–27], one reference did not have a full article available [28], and one reference reported the same data from the same program as another reference already included in our review [29].

Of the 34 studies included in our review, 30 found evidence of a reduction in health costs either to the health system or the patient, and four had a mixed impact, an increase in costs, or no changes in costs [30–33]. Almost all the studies focused on the effects of shifting clinical or public health tasks related to a specific disease or disease area, while one study focused on task shifting a HSS activity (mapping of village geographic coordinates) [34]. Only two studies examined task shifting within a hospital, whereas all others examined task shifting from the hospital to the primary health care (PHC) or community levels, or task shifting within the PHC/community level.

Of the 30 studies that found evidence of cost savings, 10 reported a cost savings per outcome, 13 reported a cost savings per output, and 3 reported a cost savings per input/process coupled with a corresponding maintenance or improvement in programmatic quality. Although cost savings on inputs/processes do not indicate efficiency improvements as strongly as savings on outputs or outcomes, the combined body of evidence from these 26 studies suggests that task shifting yields cost savings that result in efficiency improvements to the health system, especially at the PHC and community levels. The four citations which reported cost savings on an input/process and which did not report changes in clinical or programmatic quality all reported on tasks related to different disease areas/HSS activities.

The full list of references meeting inclusion criteria can be found in Table 1.

**Table 1:** Full list of citations included in systematic review

Author and year	Country	Intervention	Indicator type	Main indicator	Result	Quality data
TB						
Clarke, M., et al. (2006) [39]	South Africa	Training of lay health workers (LHWs) to support treatment and management of TB on farms, instead of clinic nurses or enrolled (non-professional) nurses	Input/process	Cost per minute of health worker time	91% reduction in cost from clinic nurses ($0.12 per minute) to LHWs ($0.01 per minute) and 87.5% reduction from enrolled nurses ($0.08 per minute) to LHWs	Farms with LHWs supporting had 42% better case finding rate and 10% better cure rate
Datiko, D. G. and B. Lindtjorn (2010) [35]	Ethiopia	Comparison of Health Facility-based DOT (HFDOT) program for TB compared with community DOT (CDOT) program using health extension workers	Outcome	Cost per successfully treated patient	63% reduction in costs from HFDOT model ($16.19) to CDOT model ($6.07)	74.8% cure rate for CDOT compared with 68.2% for HFDOT
Dick, J., et al. (2007) [37]	South Africa	Evaluation of a lay health worker project overseen by primary healthcare nurses aimed at treating TB on farms	Outcome	Cost per case detected and cured	74% cost reduction to the District Health Authority on farms with LHW program compared to control farms (absolute cost figures not reported)	Treatment completion rate for smear-positive TB patients 18.7% higher in intervention group compared to controls (*p* < .05)
Floyd, K., et al. (2003) [41]	Malawi	Community-based outpatient treatment for smear-positive pulmonary patients (instead of inpatient treatment)	Outcome	Cost per patient cured	62% reduction from hospital-based treatment ($786) to community-based treatment ($296)	Cure rate was 68% for community-based strategy and 58% for hospital-based strategy
Islam, M. A., et al. (2002) [36]	Bangladesh	BRAC TB control program using CHWs, compared to government-run program	Input/process; outcome	Total annual cost for TB control program at the subdistrict (thana) level; Cost per patient cured	31% reduction in total annual costs from government program ($10,697) to BRAC program ($7,351); 32% reduction in cost per patient cured	84.1% cure rate in BRAC TB program compared to 82.2% in government program
Khan, M. A., et al. (2002) [40]	Pakistan	Comparison of DOTS by health workers at health centers, DOTS by family members, and “DOTS without direct observation”	Outcome	Cost per case cured	45% reduction from health center DOTS ($310) to CHW DOTS ($172); unsupervised DOTS cost $164	Cure rates were 62% for unsupervised DOTS, 55% for family member DOTS, 67% for CHW DOTS, and 58% for Health Center DOTS
Okello, D., et al. (2003) [38]	Uganda	Comparison of conventional hospital-based care with community-based care for DOTS, including management by a sub-county public health worker	Outcome	Cost per smear-positive patient successfully treated	57% reduction in costs from conventional care ($911) to community-based care ($391)	Treatment success rate for smear-positive cases was 56% for conventional care and 74% within community-based care
Prado, T. N., et al. (2011) [42]	Brazil	Comparison of DOTS overseen by guardians with standard of care treatment by CHWs	Output	Total cost for DOTS course	28% reduction in costs from CHW DOTS ($547) to guardian-supervised DOTS ($389)	98% treatment completion in guardian-supervised DOTS compared to 83% treatment completion with CHW-supervised DOTS (*p* = .01)
Sinanovic, E., et al. (2003) [43]	South Africa	Comparison of clinic-based care with community-based observation by lay person with community-based care for smear-positive pulmonary and retreatment TB patients	Outcome	Cost per patient successfully treated	62% reduction in costs for new smear-positive patients from clinic-based care ($1302) to community-based care ($392); 62% reduction in costs for retreatment patients from clinic-based care ($2008) to community-based care ($766)	80% treatment success rate for community-based care, compared to 54% treatment success rate for clinic-based care
HIV						
Babigumira, J. B., et al. (2011) [46]	Uganda	Comparison of a Pharmacy-only Refill Program (PRP) to Standard of Care for treatment for HIV/AIDS patients	Output	Cost per person per year from societal and Ministry of Health perspective	21% reduction in societal costs from Standard of Care ($665) to PRP ($520) and 17% reduction in MoH costs from Standard of Care ($610) to PRP ($496)	No statistically significant difference in favorable immune response among patients in two groups
Bemelmans, M., et al. (2014) [48]	South Africa	Adherence club for ARVs led by lay counselor and offered to all clinically stable patients who had been on ARVs for greater than 12 months; Club met every 2 months for essential medical tasks (e.g., weighing and health assessment) and distribution of ARVs	Output	Cost per patient per year	46% reduction from mainstream model of care ($108) to ARV club model ($58)	<1% mortality at 40 months, and 2.8% loss to follow up at 40 months in ARV club
Fatti, G., et al. (2015) [45]	South Africa	Indirectly Supervised Pharmacist Assistant (ISPA) program compared to nurse-managed models for providing ARTs	Input/process	Human resource costs and costs per item dispensed	29% reduction in human resource costs from nurse-managed program ($1.89 per patient visit) compared to ISPA model ($1.35 per patient visit); 49% reduction in cost per item dispensed from nurse-managed program ($0.83) to ISPA model ($0.43)	Cumulative attrition lower at ISPA sites (20.7% compared to 31.5%); proportion of patients achieving virological suppression higher at ISPA sites (89.6% compared to 85.9%)
Foster, N. and D. McIntyre (2012) [47]	South Africa	Indirectly Supervised Pharmacist Assistant (ISPA) program and nurse-managed models compared to full-time pharmacist for providing ARTs	Input/process	Cost per patient visit	43% reduction in cost from nurse-driven model ($10.16) to ISPA model ($5.74) and 12% reduction in cost from full-time pharmacist model ($6.55)	
Johns, B. and E. Baruwa (2015) [31]	Nigeria	Comparison of hospital-based distribution of ART (by doctors) with clinic-based distribution of ART (by nurses and/or community pharmacists) for stable patients who had been on ART for at least 1 year, in two states aiming to decentralize health services	Output	Total cost per person per year	Total costs increased in one state by 31% and decreased in one state by 32%; In both cases, the largest difference in costs between the hospital and clinic sites was staff cost/patient visit	Few statistically significant differences found in service utilization indicators between patients going to clinic sites versus hospital sites; Patients in the state that achieved cost savings had 3.7× more visits per year than in hospitals (*p* < .01)
Johns, B., et al. (2014) [30]	Ethiopia	Comparison of minimal, moderate, and maximal task shifting for ARV responsibilities away from physicians with hospital-based ARV distribution . Minimal = nonphysicians clinicians (NPC) monitor ART; Moderate = NPC initiate and monitor ART; Maximal = NPCs initiate, monitor, treat side effects, and switch ARTs	Output	Cost per patient year	No statistically significant changes in cost/patient per year between models of task shifting or between all task shifting models and hospitals	Almost no statistically significant differences in patient retention from different levels of task shifting
Yan, H., et al. (2014) [44]	China	Evaluation of shifting HIV preventive intervention and care for men who have sex with men (MSM) from government facilities to community-based organizations (CBOs)	Outcome	Unit cost per HIV case detected	97% reduction in cost from government health facilities ($14,906) to community-based organizations ($315)	Within 4 years, total % of HIV cases reported increased from ~10 to ~50%, despite “a very low share of HIV tests by CBOs out of the total HIV tests performed each year during the pilot,” which indicates effective targeting of HIV patients for tests by CBOs
Malaria						
Chanda, P., et al. (2011) [49]	Zambia	Comparison of home management (using CHW) with facility-based management of uncomplicated malaria	Output	Cost per case appropriately diagnosed and treated	31% reduction from facility-based management ($6.12) to home management ($4.22)	100% of cases treated appropriately through home management, and 43% of cases treated appropriately in facility
Hamainza, B. M., et al. (2014) [50]	Zambia	Comparison of CHW program to test and treat malaria with facility-based testing and treatment	Output	Total cost per confirmed case treated	60% reduction in cost from facility-based approach ($10.75) to CHW approach ($4.34)	78% of CHW contacts received appropriate testing and treatment, while 53% of facility-based patients received appropriate testing and treatment based on guidelines
Mbonye, A., et al. (2008) [32]	Uganda	Community-based administration of intermittent preventive treatment (IPTp) for malaria by traditional birth attendants, drug-shop vendors, community reproductive health workers, and adolescent peer mobilizers	Output	Cost per patient of providing a full regimen of IPTp	9% increase in costs from health center care (4093 shillings) to community-based care (4491 shillings)	
Patouillard, E., et al. (2011) [51]	Ghana	Comparison of IPT administration by village health workers (VHWs), facility-based nurses working in outpatient departments of health centers or EPI outreach clinics	Outcome	Economic cost per child fully covered and fully adherent to treatment	11% reduction from using facility-based strategy ($8.51) to VHW strategy ($7.56)	69.1% of children in VHW strategy completed course, 63.8% of children in facility-based strategy completed course
Ruebush, T. K., 2nd, et al. (1994) [52]	Guatemala	Change to the supervision and distribution model of unpaid Volunteer Collaborators (VC) in the surveillance and treatment of malaria, including treatment for malaria without taking a blood smear, removal of literacy requirement for VC, and reduced supervision from once every 4 weeks to once every 8 weeks	Output	Cost per patient treated	75% reduction in cost per patient treated in modified model of VCs ($0.61) versus control network of VCs ($2.45)	Average time from examination to initiation of treatment was 6.6 days in modified model areas, compared to 14.6 days in control areas
Sikaala, C. H., et al. (2014) [53]	Zambia	Community-based (CB) mosquito surveillance and trapping using light traps (LT) and Ifakara tent traps (ITT) compared to centrally supervised quality assurance (QA) trapping teams, including human-landing catch (HLC) teams, for the prevention of malaria	Output	Cost per specimen of *Anopheles funestus* captured	96% reduction in costs from using QA-LT ($141) to CB-LT ($5.3); 83% reduction in costs from using QA-ITT ($168) to CB-ITT ($28); QA-HLC method cost $10.5	
Other diseases and health systems strengthening activities						
Aung, T., et al. (2013) [62]	Myanmar	Comparison of costs to treat diarrhea by CHW, government facility, and private provider	Input/process	Total patient cost for consultation and correct ORS	7% reduction from private providers ($5.40) to CHWs ($5) and 67% reduction from government facilities ($15) to CHWs	CHWs provided appropriate ORS and amount of drinking water in 57.6% of cases, private providers in 47.1% of cases, and government facilities in 71.4% of cases
Buttorff, C., et al. (2012) [57]	India	Comparison of “collaborative care” model using full-time physician, lay health worker (LHW), and mental health specialist with “enhanced usual care” by full-time physician only for treatment of depression and anxiety disorders	Output	Average annual cost per subject	23% reduction in costs from collaborative care model ($177) compared to physician-only care model ($229)	Patients in collaborative care improved 3.84 points more on the Revised Clinical Interview Schedule (to measure psychiatric symptoms) compared to physician-only care model
Chuit, R., et al. (1992) [60]	Argentina	Surveillance to reduce transmission of Chagas disease using Primary Health Care (PHC) agents compared to a vertically oriented program run by trained entomological professionals	Output	Cost of surveillance per house	80% reduction in cost from vertical surveillance ($17) to PHC surveillance ($3.40)	Surveillance rates and levels of infestation detection were comparable across intervention and control arms
Cline, B. L. and B. S. Hewlett (1996) [61]	Cameroon	Diagnosis and treatment for schistosomiasis by CHWs identified by the community	Output	Average cost of diagnosis and treatment of a child	90% reduction in cost from treatment at nearest pharmacy (approx. $15) to CHW model ($1.50)	7% prevalence in school children after participating in program, compared to 71% in children who did not participate in program
Fiedler, J. L., et al. (2008) [63]	Honduras	Community-based integrated child care (AIN-C) program that uses volunteers to help mothers monitor and maintain adequate growth of young children	Input/process	Cost for one child growth and development consultation	86% reduction from facility-based consultation (105.1 lempiras) to community-based program (14.67 lempiras)	
Hounton et al., (2009) [33]	Burkina Faso	Training of obstetricians, general practitioners, and clinical officers to lead surgical teams for caesarian sections	Outcome	Incremental cost of one newborn life saved	Compared to clinical officers, one newborn life saved cost $200 for general practitioners, and $3,235 for obstetricians	Higher newborn and maternal case fatality rates among clinical officers than other types of practitioners
Jafar, T. H., et al. (2011) [54]	Pakistan	Home-health education (HHE) by CHWs, home-health education plus general practitioner (GP) supervision (combined group), or general practitioner-supervision only to control blood pressure	Output	Total cost per patient over 2 years for each group	7% reduction in costs from GP-only group ($537) to combined group ($500); 27% reduction in costs from GP-only group to HHE-only group ($393)	Decline in systolic BP was highest in the combined group (*p* = .001)
Kruk, M. E., et al. (2007) [58]	Mozambique	Comparison of surgically trained assistant medical officers and specialist physicians	Input/process	Cost per major obstetric surgical procedure	72% reduction in costs using assistant medical officers ($39) compared to specialist physicians ($144)	
Laveissiere, C., et al. (1998) [56]	Cote d'Ivoire	Detection of sleeping sickness using conventional mobile teams compared to integration of activity into CHW duties	Output	Cost of surveillance per person	81% reduction in costs using CHWs ($0.10) instead of using mobile teams ($0.55)	
Puett, C., et al. (2013) [55]	Bangladesh	Community-based management of severe acute malnutrition by CHWs compared to inpatient treatment	Outcome	Cost per DALY averted	98% reduction in costs/DALY averted from observed inpatient treatment costs ($1344) to community treatment ($26) and in costs/death averted from observed inpatient treatment costs ($45,688) to community treatment ($869)	91.9% of children in community treatment area recovered, compared to only 1.4% in inpatient treatment
Sadruddin, S., et al. (2012) [59]	Pakistan	Comparison of home treatment of severe pneumonia by lady health workers with referred cases treated by other practitioners	Output	Cost per treatment of severe pneumonia	81% reduction in costs using lady health workers ($1.46) compared to referred cases ($7.60)	93.4% of cases successfully treated by lady health workers with a 5-day course of amoxicillin, and remaining cases referred for further treatment
Munyaneza, F., et al. (2014) [34]	Rwanda	Use of CHWs and nurses to collect geographic coordinates using GIS systems instead of trained and dedicated GIS teams	Input/process	Total cost of mapping activities	51% reduction in costs from using dedicated GIS teams ($60,112) to CHWs ($29,692)	

### Tuberculosis

Nine studies demonstrated cost savings with task shifting for identification, diagnosis, and treatment of tuberculosis. Strategies for reducing costs included task shifting treatment supervision to health workers in the community [35–41], to home guardians or close relatives [42], laypersons [43], and in one case entrusting patients to take medicine without direct supervision [13]. Programmatic and clinical indicators, such as treatment success rate, treatment completion rate, and case finding rate, also indicate that task shifting programs maintained programmatic quality comparable or superior to traditional models of care.

### HIV/AIDS

Studies in this review revealed cost savings from task shifting prevention and care for a high-risk group (men who have sex with men (MSM)) to community-based organizations [44], and dispensing of ART from pharmacists to Indirectly Supervised Pharmacist Assistants (ISPA), adherence clubs, or other pharmacy-only refill programs [45–48]. Programmatic indicators, such as patient retention, viral load, and mortality also indicate that these programs maintained high quality of care. These findings indicate that the dispensation of ARTs, especially to clinically stable patients who are very familiar with the routine of taking these drugs, is suitable for task shifting in low-resource (and possibly other) settings. One study examining task shifting of ART dispensation to clinics found both an increase of costs in one state and a decrease in another state [31], and one study examining the task shifting initiation and management of ART treatment found no statistically significant differences in costs [30].

### Malaria

Our review identified five articles that identified cost savings related to task shifting for malaria-related programs: CHW management of malaria [49, 50], village health worker (VHW) administration of IPT [51], community-based surveillance and treatment of malaria [52], and community-based surveillance and trapping of mosquitoes for vector control [53]. Indicators of program and clinical quality, such as administration of appropriate treatment, treatment completion rate, and average time from examination to initiation of treatment, indicate that the programs also maintained or improved programmatic quality. One study found a minor (9%) increase in the cost of administration of IPT during pregnancy when shifting to a community-based model. Although the evidence is less robust than that for TB or HIV/AIDS, these findings suggest that many malaria-related tasks can achieve cost savings from task shifting.

### Other disease areas and activities

Our review identified 11 additional studies which provided evidence of cost savings from task shifting for activities related to other diseases or health systems strengthening. These activities included controlling blood pressure through a combination of general practitioner and CHW activities [54], community-based management of severe acute malnutrition [55], integration of the detection of sleeping sickness intro routine CHW activities [56], treatment for mental health problems by a “collaborative care” team that included a lay health worker and mental health specialist [57], administration of major obstetric procedures by assistant medical officers instead of physicians [58], home-based treatment of severe pneumonia by lady health workers [59], integration of surveillance to reduce transmission of Chagas disease by Primary Health Care agents instead of specially trained professionals [60], diagnosis and treatment of schistosomiasis by CHWs [61], treatment of diarrhea by CHWs [62], community-based integrated child care using volunteers to monitor and maintain growth [63], and geo-mapping activities by CHWs and nurses instead of dedicated GIS teams [34].

## Discussion

This review aimed to identify whether task shifting can result in cost savings and efficiency improvements to health systems. Our results indicate that task shifting is a promising approach to achieving cost savings and improving efficiency in LMICs, and our results build on previous work which concluded that task shifting can be an effective way to improve population health. These findings have significant policy implications, discussed below, as well as important limitations.

1. *Task shifting can help achieve cost savings and improve efficiency for activities related to top global health priorities, emerging global health issues, and neglected tropical diseases, but the evidence base is mostly limited to PHC and community-based care*
  The most robust body of evidence found in this study is for achieving cost savings from task shifting activities related to TB and HIV/AIDS. Given the high burden of these diseases in LMICs and the longitudinal nature of preventing, treating, and managing these diseases, interventions that can reduce both their economic and health burdens simultaneously are particularly important for the future of global health. Each year there are 1.5 million new cases of tuberculosis, mostly in LMICs, and the global burden of TB amounts to approximately $12 billion annually [64, 65]. As of 2015, 36.7 million people were living with HIV, and meeting UNAIDS targets will require nearly $20 billion annually [66, 67]. TB treatment using DOTS is a relatively routine activity that occurs over many months and can take place in the community (when the infection is not drug-resistant). Dispensation of ART to clinically stable patients who know and follow their drug regimens is also a relatively routine process. Therefore, these activities are well-suited for task shifting, and health systems can likely improve their efficiency by undertaking such efforts.
  Outside of TB, HIV/AIDS, and malaria, the evidence for cost savings from task shifting was spread across many disease areas, making it difficult to conclude that task shifting activities for a specific disease could result in cost savings. Nonetheless, the fact that programs achieved cost savings from such a diverse set of diseases and across multiple geographies indicates that policymakers and program planners should consider task shifting as one of many potential approaches to improve efficiency in their health systems. The evidence for cost savings came from disease areas such as childhood illnesses, non-communicable diseases (which are receiving increased priority at the global level due to the Sustainable Development Goals), and neglected tropical diseases (NTDs).
  Almost all studies identified shifted tasks to or within the context primary health care (PHC) or community-based care. Although several citations identified cost savings by shifting tasks from hospitals to PHC or community care, only one citation found cost savings by shifting tasks within the hospital setting [58]. One additional study within the hospital setting found that shifting surgical care from physicians to clinical officers did not yield cost savings, but it did not analyze the cost-effectiveness of shifting surgical tasks from surgeons to other physicians [33]. While the body of evidence in this review suggests that task shifting can improve efficiency across multiple disease at the PHC and community levels, more research is needed on the effects of task shifting within secondary, tertiary, and highly specialized care.
2. *Models of task shifting involve more than transferring clinical care to CHWs*
  CHWs play a key role in reducing costs and increasing access to care in the health system. Nonetheless, this research shows that many models of task shifting exist outside of a simple transfer of clinical care to a CHW. Of course, many types of associate health professionals exist, such as pharmacy technicians, lay counsellors, and medical assistants, and the references included in this study reflect this diversity of health professions [68]. In particular, the use of different models for dispensing ART to HIV-positive patients was documented in multiple studies. In addition, several studies used models where CHWs or other lower-skilled workers collaborated with clinicians in order to provide a new model of care for the patient [54, 57].
  Interestingly, only two studies identified cost savings from task shifting non-clinical activities: geo-mapping by CHWs and community-based mosquito trapping and surveillance. Given the importance that many non-clinical health systems functions have on improving population health (e.g., supply chain, monitoring and evaluation), research and program planners should consider the potential that task shifting could have for other health systems-related activities. For example, it is possible that lower-skilled professionals could perform routine tasks related to monitoring the supply chain or tracking patient data without compromising the quality of the activity.
3. *The design and benefits of task shifting interventions will vary based on the context*
  Policymakers and program planners must recognize that task shifting is not a panacea for improving health and efficiency, but rather one of many tools to use in order to improve the efficiency of the health system. This review identified a range of task shifting models which resulted in different types of cost savings. Of course, without proper design, task shifting may actually increase system costs or reduce efficiency, such as by worsening overall population health due to poor clinical quality or increasing the number of staff in the health care system without changing care-seeking patterns among patients. Interestingly, one study found that the same model of task shifting resulted in both cost increases and cost decreases in two different regions of the same country [31]. Further, task shifting can also result in task overload for health workers, which could also reduce productivity and worsen health population health outcomes [69].
  The breadth of task shifting models covered in this review is consistent with other findings from the literature which also indicate the need to adapt task shifting models to local contexts and health systems. For example, one systematic review notes a number of factors which can impact the success of lay health worker programs, including acceptability of the model to patients, implementation challenges such as problems with training, and health systems bottlenecks such as challenges with payment [70]. Another systematic review specifically identified strong management of CHW programs as the most important factor in their scale-up [71]. This body of evidence therefore suggests that designing appropriate task shifting models requires a thorough investigation of the local context, disease burden, and program goals.

### Limitations of the evidence, risks, and future directions for research

There are several limitations to the research and its findings. First, this study includes citations that measure changes in cost and efficiency very differently. Of course, looking strictly at cost-effectiveness thresholds, rather than cost savings and programmatic indicators as a proxy for cost-effectiveness, would have helped to standardize these findings to make them more comparable. However, limiting our analysis to cost-effectiveness thresholds would also have negatively altered the evidence base in our review by (1) eliminating studies which demonstrated savings but did not have a formal cost-effectiveness analysis and (2) including studies that may have achieved some level of cost-effectiveness but which did not actually achieve savings (i.e., those in which an intervention by a specific cadre of health worker met a cost-effectiveness threshold). By researching the impact of task shifting on costs to the health system as a proxy measure for efficiency improvements, we have focused on a key aspect of decision-making directly relevant to policymakers.

Second, unlike systematic reviews looking at health outcomes from highly specified clinical protocols, this review cannot predict the implications of a new task shifting program. Numerous factors in a given context will affect the outcomes of task shifting, including the burden of disease, the existing human resources for health, previous task shifting efforts, the social determinants of health, and the political economy of health. We caution that researchers and policymakers should not treat this review as a guarantee that future task shifting efforts will result in cost savings; rather, they should see this review as providing compelling evidence that task shifting can achieve cost savings if there is a need for such an intervention, and it is implemented appropriately.

Third, our search only identified two citations suitable for inclusion that examined task shifting within a hospital setting. Our search did not exclude programs that delivered services at a specific level, and the search included other citations focused on hospitals or specialty care that failed to meet inclusion criteria for other reasons (see select citations for examples [72–75]). This result suggests that the absence of evidence for task shifting within hospitals is likely due to the limited research on this topic to date. Nonetheless, LMICs have implemented programs to task shift hospital-based care, such as surgical services [76, 77]. Future research should examine models of task shifting within hospitals and their impacts on health outcomes, costs, and other relevant indicators.

Finally, as already discussed, the methodology of this review is limited by biases in reporting and publication of individual references.

Going forward, we feel that researchers, program planners, and policymakers should continue to collaborate to understand both the financial and health impacts of task shifting. Many new task shifting efforts are underway globally, and ensuring that all these programs report on cost-effectiveness thresholds and changes in costs to the system will increase the evidence base surrounding this important topic. In particular, more programmatic research is needed to confirm the preliminary findings that task shifting for activities related to NCDs, NTDs, and health systems strengthening can result in cost savings, and to understand the role that task shifting can play in hospital and specialty settings. At the same time, researchers should also carefully examine the risk of task overload from task shifting and design ways to prevent and mitigate this risk.

## Conclusions

This review examined the evidence for task shifting in improving health systems efficiency in LMICs. The evidence indicates that task shifting for activities across a broad range of diseases, including TB, HIV/AIDS, malaria, childhood illness, NCDs, and NTDs, can result in cost savings without compromising clinical or programmatic quality. This review also revealed that countries have used different approaches to introduce task shifting for management of different conditions and that task shifting takes on many forms besides simply transferring clinical activities to CHWs. Going forward, researchers, program planners, and policymakers should carefully examine their local context in order to determine whether task shifting can improve health systems efficiency while also maintaining or improving population health.

## Abbreviations

ART: Antiretroviral therapy
CHW: Community health worker
DOTS: Directly observed treatment—short course
HSS: Health systems strengthening
ISPA: Indirectly supervised pharmacist assistant
LMIC: Low-income and middle-income countries
MSM: Men who have sex with men
NCD: Non-communicable disease
NTD: Neglected tropical disease
PHC: Primary health care
RMNCH: Reproductive, maternal, newborn, and child health
TB: Tuberculosis
VHW: Village health worker
WHO: World Health Organization
